# Effects of Nicotine During Pregnancy: Human and Experimental Evidence

**DOI:** 10.2174/157015907781695955

**Published:** 2007-09

**Authors:** R Wickström

**Affiliations:** Neonatal Research Unit, Department of Women and Child Health, Karolinska Institutet, Sweden

**Keywords:** Newborn, perinatal, intrauterine, programming, developmental, NRT, snuff.

## Abstract

Prenatal exposure to tobacco smoke is a major risk factor for the newborn, increasing morbidity and even mortality in the neonatal period but also beyond. As nicotine addiction is the factor preventing many women from smoking cessation during pregnancy, nicotine replacement therapy (NRT) has been suggested as a better alternative for the fetus. However, the safety of NRT has not been well documented, and animal studies have in fact pointed to nicotine *per se* as being responsible for a multitude of these detrimental effects. Nicotine interacts with endogenous acetylcholine receptors in the brain and lung, and exposure during development interferes with normal neurotransmitter function, thus evoking neurodevelopmental abnormalities by disrupting the timing of neurotrophic actions. As exposure to pure nicotine is quite uncommon in pregnant women, very little human data exist aside from the vast literature on prenatal exposure to tobacco smoke.

The current review discusses recent findings in humans on effects on the newborn of prenatal exposure to pure nicotine and non-smoke tobacco. It also reviews the neuropharmacological properties of nicotine during gestation and findings in animal experiments that offer explanations on a cellular level for the pathogenesis of such prenatal drug exposure.

It is concluded that as findings indicate that functional nAChRs are present very early in neuronal development, and that activation at this stage leads to apoptosis and mitotic abnormalities, a total abstinence from all forms of nicotine should be advised to pregnant women for the entirety of gestation.

## 1. INTRODUCTION

Although knowledge about the negative effects on the fetus and the newborn of smoking during pregnancy is getting increasingly widespread, this habit still remains a great problem worldwide. The International Child Care Practices Study concluded in a survey of 21 centers in 17 countries that an average of 22% of mothers and 45% of fathers were smoking at the time of their child’s birth [94]. Although the geographical variations are large, this most likely represents the single largest modifiable neuropharmacological exposure for the fetus. A large number of studies confirm that maternal tobacco smoking during pregnancy adversely affects pre- and postnatal growth and increases the risk of fetal mortality [3,16,66], morbidity [45,131], cognitive development [7,33], and behavior of children and adolescents [133,134].

The possible adverse health effects of smokeless tobacco during pregnancy have received far less attention with a limited number of publications addressing this practice. Characterisation of this association is however important for several reasons. Firstly, millions of pregnant women use smokeless tobacco, predominantly in Africa and Asia [40] but also in Scandinavia [58]. These women are not exposed to the combustion products in tobacco smoke (e.g. carbon monoxide and cyanide) that may contribute to fetal hypoxia and reduced birthweight, but as nicotine levels may be very high the fetal exposure to nicotine may be unaltered or even increased. Secondly, as the hazards of smoking during pregnancy have become more evident in recent years, pregnant women are in rapidly increasing numbers turning to other forms of nicotine. Therefore, the use of smokeless tobacco during pregnancy is an important clinical issue also for other countries than those primarily affected today. Finally, studying children exposed to smokeless tobacco, rather than tobacco smoke, facilitates comparison with the multitude of animal and cellular studies that have most often studied nicotine *per se*.

This article reviews current knowledge about the neuropharmacological effects of prenatal exposure to nicotine, a practise that compared to smoking is likely to be less harmful for the mother. Whether this is the case also for the fetus is however highly unclear. The article covers the current literature on studies in human infants exposed to nicotine primarily from other sources than tobacco smoke. Such data are quite scarce as use of smokeless tobacco among pregnant women is relatively less common than smoking. Furthermore, it addresses data obtained in animal experiments that allow separation of several of the confounding factors that are difficult to control in the human situation. Although the relevance of results from another species are always difficult to determine, these findings are vital for our understanding of the effects of prenatal nicotine exposure. Finally, it examines possible neural mechanisms that may contribute to the adverse effects of prenatal exposure to nicotine.

## 2. TOBACCO USE AMONG PREGNANT WOMEN

### a. Smoking During Pregnancy

It is estimated that 15 to 25% of women smoke during pregnancy [20,94,98] and although pregnancy motivates a minority of women to stop smoking for at least part of their pregnancy, most start again after delivery [98]. There is also a strong correlation between maternal smoking during pregnancy and young age, unmarried status and being from low socio-economic strata [68]. Despite current knowledge about the detrimental effects of smoking during pregnancy, the reduction in smoking among pregnant women is progressing very slowly. In some regions, e.g. Sweden, smoking has decreased at a higher rate and is now below 10% [125]. This decrease is however often coupled to a simultaneous increase in the use of smokeless tobacco, resulting in virtually unaltered levels of nicotine exposure for the fetus [125].

### b. Other Sources of Nicotine

It is well established that nicotine replacement therapy (NRT) increases the likelihood of successful smoking cessation in non-pregnant adults by 1.5 to 2-fold, regardless of what (if any) other efforts are made [112]. Whether this is also true for pregnant women is however unclear, and is discussed further below. Although this is surely beneficial for the woman’s health, it is unclear if the offspring is any better off with this strategy. The main mode of action of NRT is thought to be the stimulation of nicotinic receptors in the ventral tegmental area of the brain and the consequent release of dopamine in the nucleus accumbens.

In clinical praxis, pregnant women in most countries are advised to avoid any form of nicotine exposure if this is possible [95]. However, there is a general view that NRT during pregnancy is safer than smoking, recently reflected in a British Medical Journal editorial entitled “Nicotine replacement therapy in pregnancy is probably safer than smoking” [20]. NRT in pregnancy is therefore being encouraged in the absence of any direct evidence for its safety. Around 30% of pregnant women succeed in stopping smoking during pregnancy without pharmacological support, but for those who do not succeed, or have previously failed in an attempt to quit, the use of NRT to support smoking cessation in pregnancy is often considered justifiable in relation to the risk of continued smoking [85]. Recent studies also confirm that nicotine replacement therapies are commonly prescribed or recommended to pregnant smokers by obstetric providers. Interestingly, pediatric clinicians were more reluctant to NRT prescription [97].

As knowledge about the adverse effects of maternal smoking during pregnancy has increased over the last 15 years, women in several parts of the world have turned to alternative sources of nicotine. Similarly, smoking among women in South East Asia is rare, but use of smokeless tobacco is common [103]. Among women in India, smokeless tobacco has been demonstrated to constitute more than 95% of the total tobacco consumption, thereby by far surpassing smoking as the primary source of nicotine during gestation [40]. In the Mumbai Cohort study [41], 58% of 59,527 lower-middle and lower-class women age 35 years and old reported current tobacco use, virtually all of which was smokeless.

A possible confounder of all studies of tobacco use during pregnancy, and in particular those emanating from developing countries, is that tobacco use tends to be associated with several other risk factors. In India, women using smoke-less tobacco had relatively lower socioeconomic status, weight, and educational status and were less likely to have had optimal antenatal care [44]. Similar associations are likely to occur in most countries and need to be considered when interpreting results.

## 3. PHARMACOLOGICAL ACTIONS OF NICOTINE

The causative agent for the adverse effects of maternal smoking during gestation have been difficult to elucidate as cigarette smoke contains thousands of biologically active compounds, some of which are known to be fetal toxins (e.g. carbon monoxide (CO), metals and nicotine). 

The adverse effects on the fetus of maternal smoking are likely to be multi-factorial, including indirect effects such as poor nutritional status of the mother associated with the anorexigenic effect of nicotine, carbon monoxide exposure, and blood flow restriction to the placenta due to the vasoconstrictive effects of catecholamines released from the adrenals and nerve cells after nicotine activation. Direct effects on nicotinic acetylcholine receptors (nAChRs), which are present and functional very early in the fetal brain [5] are also likely to contribute. 

Due to the pH of the cigarette smoke, only small amounts of nicotine are absorbed through the buccal mucosa [38]. In contrast, nicotine is rapidly absorbed when the tobacco smoke reaches the small airways and alveoli of the lung. This causes a quick rise in blood nicotine concentrations, but due to the eventual burnout of the cigarette, these levels also peak early and thereafter drop to lower levels [10]. Alternative sources of nicotine can be divided into two distinct groups: a) pure nicotine products (nicotinic patches, gum, oral inhaler, nasal spray, lozenges or sublingual tablets), and b) smokeless tobacco (chewing tobacco, oral moist snuff and mishri).

Comparison of typical steady-state plasma nicotine concentrations from different modes of administration has proven difficult. Whereas some studies report similar levels as cigarette smoking (typically ranging from 10-50 ng/ml) [96], others report substantially higher [81] or lower [57] levels of nicotine. One reason for this discrepancy is different definitions of how to define “levels” of nicotine. Although the rise in the plasma and brain nicotine levels are slower using NRT than with smoking, concentrations of nicotine in the blood also decline much slower [12,57]. The total dose of nicotine (i.e. the area under the curve) is therefore in practise likely to be higher in persons using these formulations than after smoking a cigarette. The exception is nasal spray which provides a rapid delivery of nicotine almost similar to that seen after smoking [39,129]. Consequently, the risk of developing addiction has been demonstrated to be higher in subjects using nasal spray as compared to other forms of NRT [135]. The overall risk was, however, much lower than for those using cigarettes. 

An estimate of blood levels of nicotine with nicotine patches range from 10 to 20 ng/ml, and 5 to 15 ng/ml for nicotine gum, inhaler, sublingual tablet, and nasal spray [9,11,110](for review of kinetics see [57]). From these data it has been estimated that ad libitum use of smokeless tobacco results in one-third to two-thirds the concentration of nicotine that is achieved by cigarette smoking. Oral snuff contain concentrations of nicotine similar to cigarette tobacco, whereas chewing tobacco only contain about half of the concentration [132]. These formulations are also buffered to alkaline pH to enable oral absorption and particularly moist snuff is capable of rapidly delivering high doses of nicotine [28]. Using nicotine patches, nicotine base is slowly absorbed through the skin, appearing in the bloodstream after about one hour. There is also a deposition in the skin, causing a continued absorption after removal of the patch corresponding to about 10% of the total dose [29].

The plasma half-life of nicotine is about 2 h, provided a low level of first-pass metabolism. However, conflicting results exist and if urinary excretion of nicotine is used the terminal half-life averages 11 h [59]. This discrepancy may be partly explained by the fact that the latter method is considered more sensitive to lower levels of nicotine and that nicotine accumulates in body tissues. Therefore, although peak and trough levels follow each cigarette, basal nicotine levels rise during the day and the influence of peak levels may be of less importance [10]. This is of particular importance when discussing NRTs as these lack the typical peak and trough levels one would expect in cigarette smokers. After absorption, the distribution of nicotine in the body is rapid and has a particularly high affinity for the brain, heart and lungs. Nicotine’s primary metabolite is cotinine, but metabolisation also occurs by glucoronidation and *N-*oxi-dation [57]. Due to the relatively longer half-life of cotinine (17 h *vs*. 2 hours for nicotine in non-pregnant adults), this compound is often used as a marker of smoking as it is more stable and therefore easier to measure.

Nicotine exerts its effects by binding to nicotinic acetylcholinergic receptors (nAChRs), located in the brain, in autonomic ganglia, the adrenal medulla and the neuromuscular junctions. Several subtypes of nicotinic cholinergic receptors exist, composed of different subunits, and activation of these receptors affects the release of a multitude of neurotransmitters including dopamine, noradrenaline, adrenaline, acetylcholine, serotonin (5-HT), γ-aminobutyric acid (GABA), glutamate and neuromodulators such as substance P. Compared with the amount of data of the pharmacological dynamics of nicotine and cotinine in non-pregnant adults, less is known about the situation during pregnancy. There are, however, evidence that nicotine is metabolised more quickly in pregnancy. Plasma clearances of nicotine and cotinine are increased by 60% and 140%, respectively, and the half life of cotinine is reduced in pregnant women (9 h v 17 h in non-pregnant women) [22]. This indicates that the correlation between plasma levels of cotinine and intake of tobacco cannot be automatically transformed from the non-pregnant to the pregnant adult.

To date, however, the efficacy of NRT therapy in pregnancy is not known as evidence comes from only two studies and is inconclusive. A randomised study failed to demonstrate an increased likelihood of smoking cessation using nicotine patches, but the numbers studied were small, and the trial was underpowered to actually determine whether nicotine replacement was effective [138]. The second study was not placebo controlled and any effects by NRT were difficult to discriminate due to the experimental design [50]. It should be noted, however, that there are at present no studies that have demonstrated positive effects of NRT in pregnant women. Also, these findings may reflect that pregnancy in itself is a very potent motivation for smoking cessation and as nicotine is metabolised more rapidly during pregnancy [22], higher doses than conventional NRT may be needed to obtain an effect.

When considering the potential fetal toxicity of any given drug, the disposition between the mother and the fetus is of vital importance. Nicotine easily crosses the placental barrier, and in humans it can be detected in the fetal circulation at levels exceeding maternal concentrations by 15%, while amniotic fluid concentrations of nicotine are 88% higher than maternal plasma [75,99]. This transfer is rapid with peak concentrations in the fetus after 15-30 minutes [75]. Nicotine also accumulates in breast milk, extending the nicotinic exposure to the postnatal period during breastfeeding [21,74]. Similarly, an accumulation of nicotine in the fetal brain has been demonstrated in rats [109].

## 4. EFFECTS OF NICOTINE IN HUMANS

### a. Effects on Pregnancy and the Newborn

The first suggestion of an association between maternal smoking and preterm labour and delivery came in 1957 and identified a premature birth-rate (defined as birthweight <2500 g) nearly twice that of non-smokers. Also, a dose–response relationship of cigarette smoking and premature delivery was described [114]. This association has thereafter been confirmed in a number of studies, but data have also given conflicting results. A recent study offers a possible explanation to the discrepancy as it indicates that maternal smoking in fact lowers the risk of very preterm birth due to gestational hypertension, but increases the risk of very preterm birth due to other mechanisms [15]. This complexity may also explain why the effects of maternal smoking are connected to the parity of the mother. Also in a study from the United States, the association between smoking and preterm delivery before 33 weeks’ gestation was stronger than for later preterm delivery [111]. 

Women using smokeless tobacco have also been shown to give birth earlier than women not using tobacco (mean difference 6.2 days), increasing the risk of preterm birth by a factor of 1.4. The crude relative risk for birth before 32 weeks was 3.7 and for birth before 28 weeks it was 7.2 [42]. Similar findings were seen in a study from South Africa, where snuff users had a significantly shorter gestational age than the both smokers and control subjects [128]. However, interpretation of this study is limited as data are limited and the demographical base is extremely complicated with a very high risk for confounding factors.

A number of studies have investigated the cardiovascular effects of nicotine during pregnancy, these have been reviewed extensively elsewhere [23]. The conclusion of these studies is that NRT appears to have cardiovascular effects on both the mother and the fetus, but that the effects are rather small. Most of the studies deal with the effects of acute nicotine exposure, and will therefore not be further discussed in this article. It is, however, important to notice these findings as they point to other factors than uteroplacental insufficiency as being responsible for the detrimental outcome of nicotine during pregnancy. The hypothesis that other constituents of tobacco smoke than nicotine affect the cardiovascular system is corroborated also by recent findings from a study of all live births in Sweden 1999-2000. Whereas pre-eclampsia was reduced in smokers (adjusted odds ratio, 0.63) it was increased in snuff users (adjusted odds ratio, 1.58) [25]. This indicates that the protective factor against pre-eclampsia in cigarette smoke is a product of combustion rather than nicotine. In the absence of this protective factor, nicotine may in fact raise the pre-eclampsia risk through effects on the cardiovascular system, such as by causing endothelial dysfunction or raising blood pressure. Although it cannot be ruled out that additives in snuff (e.g. sodium and liquorice) cause an elevation in blood pressure, this is unlikely as most brands of Swedish snuff do not contain liquorice and salt restriction has not been found to prevent pre-eclampsia [78].

An association between maternal smoking and a reduced birth weight is also well established, displaying a clear dose-response pattern. In fact, between the years 1957 and 1986, over 100 studies (comprising more than 500 000 births) were published demonstrating that children born to smoking mothers have lower birthweights [76]. A review of pooled data concluded that the decrease in mean birthweight in infants of smoking mothers was 187 g (range 70–242 g) [1]. The effect is however only seen if smoking is continued past gestational week 30, coinciding with the maximum growth of the fetus [49].

Several studies from India have demonstrated a relationship between smokeless tobacco use during pregnancy, mainly mishri (a pyrolysed and powdered tobacco), and effects on birthweight. Of 1388 singleton births in a Pune hospital, tobacco chewers had babies with a consistent birth weight deficit of 100-200 g, independent of maternal weight, socioeconomic status, and gestational age [67]. In another Indian study from the city of Mumbai of 178 deliveries, the proportion of low birth weight babies in users was twice as high as that of non-users [82]. A prospective, population based study from the same region corroborated these findings with babies born to mothers using smokeless tobacco being on average 105 g lighter [42]. When adjusted for gestational age, the difference was 87 g with a significantly larger decrease in boys than girls. This study also demonstrated a dose-response relationship when comparing light users (mean decrease 63 g) and heavy users (mean decrease 189 g). In contrast, babies of mothers using dry snuff in South Africa displayed only a non-significant decrease in birth weight (29.4 g), whereas those born to smoking mothers displayed a significantly lower mean birthweight (165 g) [128]. This discrepancy as compared to the studies from India may be due to differences in type of tobacco and possibly lower blood nicotine levels as the amounts of snuff was not recorded. Also, as mentioned earlier, this study is limited by a complex demographical base with a very high risk for confounding factors.

In a large study from the Swedish Birth Registry of 789 pregnant women using snuff, 11,240 smokers, and 11,495 nonusers, a relatively smaller effect on birth weight as compared to smoking mothers was also seen. Compared with nonusers, the adjusted mean birth weight was reduced in snuff users by 40 g, in light smokers by 172 g, and in moderate-to heavy smokers by 224 g. As the registry contained mothers whose late gestational tobacco use was not known (and therefore may represent quitters), the study population was also restricted to women whose late pregnancy tobacco exposure was known The adjusted mean birth weight was then reduced in snuff users by 93 g, in light smokers by 213 g, and in moderate-to-heavy smokers by 250 g [25]. This is a large study and the findings clearly suggests that other factors in smoking, e.g. carbon monoxide, play a more prominent role than nicotine in fetal growth restriction among smokers. However, one major drawback of this study is that the amount of snuff was not possible to extract from patient records, potentially leading to an underestimation of nicotine exposure.

In a trial of NRT in pregnancy, babies born to women in the nicotine treatment group had significantly higher birth weights than those in the placebo group (mean difference 186 g) [138]. This again may indicate that other factors than nicotine (e.g. CO or effects on the placenta) may be responsible for the growth retardation seen in children of smoking mothers, but also that the levels of nicotine is likely to be of vital importance for possible adverse effects. It has been thought that the main effect of nicotine on gestational parameters is caused by a vasoconstriction of uteroplacental blood vessels, leading to a reduced blood flow to the placenta and a subsequent reduction in the delivery of oxygen and nutrients to fetus. However, studies of blood flow in humans have failed to demonstrate a correlation between such a uteroplacental insufficiency and fetal growth retardation, except when blood flow is severely compromised (reviewed in [23]).

The neurological consequences of a moderately reduced birth weight are not as clear as those of prematurity or of a more pronounced decrease in birthweight [56,130]. A study of IQ in 6-years old children has demonstrated a correlation between low birthweight and a lower IQ score, also for those with a more moderate decrease in birth weight [14]. However, animal studies (further discussed below) indicate that birth weight is a poor marker of neurological outcome and that neural consequences may occur at much lower doses of nicotine than those required for reducing birth weight in the offspring.

Another important finding from several studies is that smokeless tobacco appears to increase the rate of stillbirth. The association was described almost 30 years ago, where tobacco-chewing mothers in India were found to have a rate of stillbirth 3 times higher than that in nonusers [67]. In western countries, maternal cigarette smoking has been causally associated with an increased risk for stillbirth; the relative risk ranges from 1.2 to 1.6 [2]. A recent study from India also reports in increased risk for stillbirth among users of NST, most of whom used mishri. After adjustment for confounding factors, the risk for stillbirth in users was 2.6 with a dose–response relationship between the daily frequency of use and stillbirth risk. The risk of stillbirth was also greater in earlier gestational periods [43]. Use of Smoke-less tobacco use during pregnancy thus appears to increase the risk of stillbirth at least as much as does maternal cigarette smoking.

The effects of maternal smoking during gestation on the risk for perinatal morbidity is well demonstrated, showing an increased risk in a dose-response fashion. This is illustrated by studies of children born to mothers who smoked 20 cigarettes or more, displaying a lower 5-minute APGAR scores and umbilical pH level, and more than 50% of these newborns required intensive care at the NICU [45,131]. Also, the perinatal mortality rate among smokers is 150% greater than is seen in non-smokers [66]. No similar studies on perinatal morbidity exist to date for NST, with the exception of the rate of stillbirth discussed above and neurobehavioural effects discussed below.

### b. Neurobehavioural Effects

The neurobehavioural effects of in utero exposure to tobacco *via* maternal smoking has been reviewed elsewhere [26]. In summary, the data indicate that in utero exposure to tobacco is associated with motor, sensory, and cognitive deficits in infants and toddlers, suggesting a toxic effect of tobacco on early neurodevelopment. Similar findings were seen in older infants where maternal smoking also was associated with a significant increase in externalising (e.g., oppositional, aggressive, overactive) behavioural problems. The behavioural and cognitive deficits associated with in utero exposure to tobacco thus seem to continue into late childhood and adolescence and to lead to increased risk for attention-deficit/hyperactivity disorder (ADHD) and conduct disorder (CD). No similar studies exist after *in utero* exposure to smokeless tobacco, leaving the question of what etiological agent(s) of tobacco smoke are mediating these effects open. However, animal studies (as described further below) indicate an important role for nicotine in this pathogenic process.

Infants born to smoking mothers are also at increased risk for developing nicotine dependence later in life. Signs of abstinence and altered behaviour are clearly visible in the perinatal period and follow a dose-response relationship [37,70]. During adolescence, a greater than 5-fold increased risk of adolescent-onset drug dependence was seen in girls whose mothers smoked 10 or more cigarettes almost daily during pregnancy. These findings could not be explained by maternal substance abuse during pregnancy, parental psychiatric diagnosis, family risk factors, prenatal and early developmental history of offspring, postnatal maternal smoking, or smoking in the offspring [134]. A direct specific action by nicotine on the developing human brain is possible during a large part of the prenatal life, since the nicotinic receptors are already present in the brain during the first trimester [52].

### c. Autonomic Effects and SIDS

The association between the risk for Sudden Infant Death Syndrome (SIDS) and maternal smoking during pregnancy is well established and smoking is the strongest risk factor for SIDS displaying a definite dose–response relationship. Maternal smoking of more than 20 cigarettes per day increases the relative risk for SIDS five-fold [84]. This association was further strengthened following the “back to sleep”-campaign, advocating a supine sleeping position for all newborns. Whereas this reduced the incidence of SIDS by about 50%, it increased the association with smoking, making it the sole most important risk factor [24,54]. It has been estimated that approximately 30–40% of all cases of SIDS could be avoided if all pregnant women stopped smoking in a population with 30% pregnant smokers [138].

As the mechanism behind SIDS is not fully understood, nor has the mechanism for the increased risk by maternal smoking been elucidated. However, concordant with the hypothesis of an altered arousability in SIDS victims, such a deficiency has been demonstrated in children of smoking mothers [55]. Similarly, it has been demonstrated that hypoxic arousal was reduced after maternal smoking [72]. Measuring autonomic function in human infants is quite complicated as many deficiencies are not visible under basal conditions. Therefore, indirect evaluation of the autonomic nervous system is often employed, using either challenge tests involving measurements of heart rate and blood pressure responses after stimulation of peripheral chemoreceptors or baroreceptors, or by power spectral analysis of heart rate variability (HRV). In children of smoking mothers, an increasing number of cigarettes was correlated with deeper the heart rate (HR) declines and smaller HR rises. Also, HR response lag time after the ventilatory response to hyperoxia, hypoxia and hypercapnia was 2.5 s greater than that in the control group for all three stimuli., This difference also increased with the number of cigarettes smoked by the mother in a dose-dependent manner [127]. 

Similarly, effects on the resting spectral power pattern of heart rate during different sleep stages was seen in infants of smoking mothers, studied at a median postnatal age of 10.5 weeks. A decreased HRV was seen at both ends of the spectrum, resembling the effects of long-term smoking in adults [32].

It has also been demonstrated that SIDS victims have a deficit of 5-HT receptors in areas in the medulla oblongata, containing abundant serotonergic projections to the respiratory system that regulate defense mechanisms to exogenous stressors such as asphyxia. A decreased serotonergic receptor binding was seen in the arcuate and caudal raphe nuclei in the majority of the SIDS victims examined [64]. This may result in failure of protective responses to life-threatening stressors such as asphyxia, hypoxia, or hypercarbia. Recent studies by the same group has further described the abnormalities in both 5-HT neuron firing, synthesis, release, and clearance. Male SIDS cases also had slightly lower 5-HT (1A) binding density in the raphe obscurus as compared with female cases, thus suggesting a neurochemical gender difference that may help explain the increased vulnerability of boys to SIDS [100].

## 5. EFFECTS OF PERINATAL NICOTINE IN ANIMAL MODELS

### a. Effects on Gestation and the Newborn

As with humans, studies in monkeys and sheep demonstrate that acute nicotine exposure has cardiovascular effects on both the mother and fetus. However, these effects require extremely high doses of nicotine and do not support the uteroplacental insufficiency model [65,86,104]. Using osmotic pumps during gestation, it has been demonstrated in a number of studies that whereas chronic nicotine exposure during pregnancy reduces maternal weights, it usually does not affect fetal growth [92,101,121]. In other studies, signs of gestational toxicity in the form of fetal resorption and intrauterine growth retardation are described [35,90,113,120]. However, in these studies a dose of 6 mg/kg*day free base was used, that may lead to maternal mean nicotine levels of approximately 80 ng/ml [73,87] and thus exceed the levels found in the most heavy smokers. In a study which compared the effects of nicotine and epinephrine it was concluded that both had similar effects on uterine blood flow and reduced maternal weight gain without affecting fetal growth (Birnbaum 1994). It therefore appears that fetal birth weight is only moderately affected by a dose of 6 mg/kg*day nicotine bitartrate, and has no effect at lower doses.

Studies of gestational length in animals are scarce, possibly because the relatively shorter gestational periods in rodents blunt results. However, there are studies that have indicated a increased variability in the timing of birth in rat pups prenatally exposed to nicotine [105]. It is important to note that lowering the dose of nicotine in rats to the point where growth impairment vanishes, and where plasma levels match those of moderate smokers, still produces all the signs of fetal brain damage that are seen at higher doses [116]. This has high clinical relevance since intrauterine growth retardation is the most commonly used predictor of adverse perinatal outcome in offspring of smokers [24].

### b. Neurobehavioural Effects

Although evidence for a deleterious effect of in utero exposure to nicotine on behaviour and cognition later in life seems overwhelming, it is difficult to separate these effects from other confounding environmental and genetic factors, e.g. parental characteristics such as IQ, maternal health, family psychiatric history and substance abuse as well as smoking characteristics. For this reason, the use of animal models, which permit the control of environmental factors, becomes critical to validate findings in humans. However, especially in the case of neurobehavioural effects, the generalization of findings of animal studies to human function requires caution, given species differences and methodological limitations.

Enhanced locomotor activity is associated with in utero nicotine exposure in several species (e.g. rats, mice, and guinea pigs) [4,34,80]. Such enhanced locomotor activity has been considered to support the association with ADHD [83], but a direct link is difficult to establish due to the non-specific nature of the changes in locomotor activity in animals. 

Also, cognitive impairments after prenatal exposure to nicotine has been demonstrated in animals. Doses as low as 0.5 mg/kg nicotine per day injected during pregnancy, caused selective deficits in learning an avoidance response in 2-month old male rats [36]. The cognitive effects after nicotine exposure in utero also seem to stretch into adulthood. Attention and memory deficits in performance on various maze tasks were seen in adult offspring of nicotine-treated rats and mice (1.5–6.0 mg/kg nicotine daily) [71,126,141]. These impairments in attention, memory, and learning are also considered consistent with the cognitive deficits found in psychiatric disorders such as ADHD.

Not all findings are positive, however, and several studies fail to report neurobehavioural effects after in utero nicotine exposure [13,102]. This may reflect differences in experimental methodology, but also that the cognitive effects are of a relatively small magnitude. In addition, it emphasises the great difficulty in performing behavioural studies with the high demands put on the experimental conditions. Understanding the neuropharmacological mechanisms of action of nicotine is essential for understanding the behavioural consequences of prenatal exposure to nicotine.

### c. Autonomic Effects and SIDS

The effects of prenatal nicotine exposure on autonomic function has been investigated in several different species (for review, see [46]). These indicate that the resistance to severe hypoxia or anoxia is impaired by prenatal nicotine exposure, most likely due to a deficient sympathetic activation during hypoxic stress leading to an inadequate respiratory adjustment. These results also correspond well to results obtained in infants of smoking mothers. Whether these effects are primarily due to direct effects on the cardiorespiratory control system, or rather reflect the extensive effects on brain and synaptic transmission by prenatal nicotine exposure has not been clearly established. The profound effects of prenatal nicotine exposure on central and peripheral catecholaminergic pathways are however likely to be involved. Decreased levels of catecholamine synthesizing enzymes in the brain and adrenals [137] and a decreased release of catecholamines [118] may be deleterious for an organism during e.g. hypoxic stress and is hypothesized to be involved in the pathogenesis of SIDS. It has also been demonstrated that the hypoxic defence mechanisms in prenatally nicotine-exposed lambs was not further compromised by postnatal nicotine exposure [48]. This indicates that the detrimental effects on this defence mechanism is established prenatally.

As with human infants, a delayed arousal during acute hypoxia has been found in prenatally nicotine-exposed 5-day-old lambs [47]. This effect was seen using a low maternal nicotine free base dose (0.5 mg/kg*day) resulting in an average maternal nicotine concentration of 7 ng/ml.

An appropriate response to asphyxia includes both arousal and autoresuscitation reflexes such as gasping, and a functional autoresuscitory system is vital for survival during severe or prolonged hypoxemia. Since defective defence mechanisms are considered to be part of the pathogenesis of SIDS, a deficient autoresuscitation may also be expected in animals prenatally exposed to nicotine. This was examined in newborn rats using a nicotine tartrate dose of 6 mg/kg*day in the pregnant dam. Whereas there was no effect of prenatal nicotine exposure on the duration of gasping in anoxia at an age of 5 or 6 days, the ability to repeatedly autoresuscitate after periods of was reduced in nicotine-exposed animals [30]. The same group also determined the threshold level of maternal nicotine tartrate that could impair protective responses, and found that 3 or 6 mg/kg/day impaired the ability to autoresuscitate from intermittent hypoxia-induced primary apnea but a dose of 1.5 mg/kg/day had no effect [31]. This may be part of the underlying mechanism resulting in increased mortality seen among nicotine-exposed rat and mouse pups during severe hypoxia [120,137].

The response to hypoxia also appears to be partially regulated by a nAChRs. In newborn transgenic mouse pups lacking β2-containing nAChRs, deficits usually associated with maternal tobacco and nicotine use, i.e. growth restriction, unstable breathing, and impaired arousal and catecholamine biosynthesis were demonstrated [19]. This indicates that the underlying mechanisms of nicotine's detrimental side effects on a range of crucial defensive reflexes involve loss of function of nAChR subtypes, possibly *via* activity-dependent desensitization.

### d. Limitations of Animal Models

Although the detrimental effects of prenatal exposure to tobacco smoke have been demonstrated in a large number of epidemiological studies, determining the underlying biological mechanisms for this association has proven difficult. This is partly caused by the lack of relevant physiological and pharmacological models in which this may be studied, as the human situation contains numerous confounding factors that are sometimes hard to control. To establish a relevant animal model, simulating maternal smoking, some investigators have performed studies in which the pregnant dams were exposed to environmental tobacco smoke. Although this experimental set-up resembles the human situation, results may be affected by the fact that the animals are likely to be stressed by the smoke complicating interpretation [61,124]. It is in this aspect similar to other previous attempts of providing nicotine during gestation such as *via* subcutaneous injections. Similarly, administration of nicotine *via* water or food has complications as it leads to dehydration or anorexia of the pregnant mothers.

Instead, most animal models to date have employed nicotine as a substitute for tobacco smoke, usually as a continuous infusion from an osmotic pump [73,87,91,93,123,137]. Although this enables comparison of total levels of nicotine, this mode of administration does not fully resemble the normal peaks of plasma nicotine levels seen in smokers [10,57]. It does, however, eliminate the hemodynamic stress on the developing fetus that the previously used injections of nicotine caused [62,119,122]. Osmotic pumps deliver a constant amount of nicotine, thereby more resembling the delivery mode seen in women using nicotine patches or oral snuff than smoking. In most studies, the administered doses range from 1 to 6 mg /kg*day of nicotine bitartrate, calculated for the initial weight of the dam. However, it is sometimes unclear in study protocols if the dose given is indeed nicotine bitartrate or free base nicotine, which constitutes 35% of the nicotine bitartrate dose. The commonly used dose of approximately 6 mg/kg*day nicotine bitartrate, corresponding to 2.1 mg/kg/day free base nicotine, achieves maternal mean nicotine blood levels of approximate 35 ng/ml. To set this in the human perspective, it has been demonstrated that a dose of 1.5 mg/kg per day produces a plasma nicotine concentration in rats similar to that of humans who smoke about 20 cigarettes per day [87]. Comparison however requires many factors to be taken into account, e.g. the increased nicotine metabolism and differences in receptor levels between species. The plasma half-life of nicotine in the mouse is 5–7 min and in the rat about 54 min. Therefore, higher doses may be required to elicit the same effects in the these species [106,115,117]. Also, the difference in neural maturity at birth across species complicates comparison. For example, cerebral maturity at birth in humans approximates that of a 10-day-old rat but this difference is not likely to be even across different brain areas as these develop at different times.

## 6. NEURAL MECHANISMS OF NICOTINIC ACTIONS

It has long been known that neurotransmitters during development have trophic roles in the cellular and architectural development of the central nervous system [17]. Therefore, activation of neurotransmitter receptors during development may induce effects others than those seen in a mature nervous system, e.g. promotion of neural cell replication and differentiation, induction (or prevention) of apoptosis, and affect neuronal migration in the brain. Is also follows from this that biological compounds that interfere with these developmental roles may interfere with the formation of the nervous system, a process that occurs well beyond the time of birth. As acetylcholine acts as a trophic factor in brain development, nicotine from maternal smoking may be expected to interfere with neurotransmitter function and evoke neurodevelopmental abnormalities by disrupting the timing or intensity of neurotrophic actions. Such disruption may be expected to have long-term consequences [91].

As mentioned above, findings from human smoker do not obligate an underlying cholinergic mechanism, as cigarette smoke contains thousands of bioactive compounds. Therefore, animal models will help our understanding on the specific role of nicotine needs in this process.

### a. Nicotinic Receptors During Development

Our knowledge about the structure and function of nAChRs comes primarily from studies in animals (mainly rodents and chicks). There are, however, differences between species where not all subunits are expressed in mammals and the developmental course of nAChRs differs across species. Generalization to humans of findings in animals thus needs to be done with caution. 

The importance of nAChRs and of nicotinic signaling in neural development is reflected in the variation in the distribution in the brain with the developmental stage and age. Studies in human fetuses have shown that mRNA expression of subunits α3-5, α7 and β2-4 of the nAChR is present in the prenatal spinal cord, medulla oblongata, pons, cerebellum, mesencephalon, subcortical forebrain and cortex during first trimester development, even as early as gestational week 4-5 [51]. This suggests an important role for nAChRs in modulating dendritic outgrowth, establishment of neuronal connections and synaptogenesis during development. Recent findings in rodents also indicate that the nAChRs are functional very early in brain development [5].

It is well established that prenatal exposure to nicotine increases nAChR density in regions of the fetal and neonatal human [27,53] and rat brain (Hagino and Lee, 1985; Sershen *et al*. 1982; Slotkin, 1998; Van de Kamp and Collins,1994) and that this effect may last beyond the neonatal period [116]. It is unclear whether this reflects a compensatory up-regulation that may be expected in a mature brain, or rather is a consequence of a disturbed cellular development. Such a disruption may either be a direct effect on nAChRs, or indirect *via* e.g. modulation of NE and DA release which act as neurotrophic factors [118]. Up-regulation of nAChRs following prenatal nicotine exposure also occurs at low doses, insufficient to cause fetal growth retardation [92]. The long-term effects of nAChR up-regulation in the fetal brain are however difficult to elucidate, as numeric up-regulation of nAChRs is in fact rather associated with a down-regulated function [77,139]. Therefore, nAChR up-regulation may cause an initial hypersensitivity followed by subsequent functional down-regulation. This type of paradoxical effect may be part of the explanation for the findings in transgenic mice lacking certain nAChR subtypes that resemble animals prenatally exposed to nicotine [18,19,77].

The hypothesis that the detrimental effects on CNS development largely depends on activation of nAChRs is supported by findings that areas more dense in such receptors display the largest degree of cellular damage [116]. However, alterations are seen also in areas that are relatively sparse in nAChRs, such as the cerebellum [122]. This may partly reflect that effects are also mediated by presynaptical activation of nAChRs and *via* other transmitter systems, but also emphasizes the importance of acetylcholine during ontogeny.

### b. Developmental Disruption

Acetylcholine has vital functions in virtually all phases of brain maturation (recently reviewed by [117]), influencing development as early as gastrulation [69]. This effect may well be mediated by nAChRs, as there are present and functional receptors in the early neural tube stage [5]. Later during development, acetylcholine promotes the switch from replication to differentiation [116] and modulates synaptogenesis [91]. Direct actions of nicotine on the fetal brain have been shown to induce abnormalities in cell proliferation and differentiation, leading to regionally specific abnormalities in cell number and macromolecular content [89,119].

Nicotine has also been demonstrated to in itself produce brain cell damage, as indicated by elevated levels of biomarkers of cell damage (e.g., ornithine decarboxylase activity) and decreased levels of DNA [123]. As a continued decrease in the number of cells was seen until postnatal week 2, it was interpreted as a change in the program for the cellular development. This is in line with the concept of perinatal or developmental programming, i.e. a lifelong alteration in genomic expression and cellular function in response to events during vulnerable periods such as perinatal life [6]. Concordantly, a reduction in brain weight and cortical thickness, as well as a reduced cellular size lasting until postnatal day 40, was seen in rats prenatally exposed to nicotine [107]. Findings of increased levels of the primary response gene c-fos in brain regions of animals prenatally exposed to nicotine suggests involvement in the regulation of apoptosis [118]. The constitutive activation of genes associated with apoptosis also coincides with the period of maximal cell loss [116]. Interestingly, the gross dysmorphology in the brain caused by nicotine exposure, is partly overcome even when nicotine administration continues through parturition, leading to a relatively normal brain morphology when examined later on in adolescence or adulthood [108]. However, because the normalization of DNA content occurs during gliogenesis, it is likely that glial cells and not neural cells replaced missing cells. This nicotine-induced apoptosis in the developing brain thereby contrasts to the proposed neuroprotective effect in the mature brain [60,63] emphasizing the importance in considering the developmental context. The involvement of acetylcholine and nicotine in regulating neuronal apoptosis also extends the dependence upon trophic responses to acetylcholine into the relatively late phases of brain development including infancy and adolescence [8]. This may be especially crucial for the central cholinergic pathways that control learning and memory [79,88].

### c. Effects on Other Neurotransmitter Systems

In addition to giving rise to structural changes, nicotine also alters neurotransmitter release, presumably *via* a presynaptic nAChR modulatory effect. Studies of brain slice preparations have indicated an enhanced presynaptic release of ACh, dopamine (DA), noradrenaline, 5-HT, g-aminobutyric acid, and glutamate [35,140]. Also post-synaptically, prenatal nicotine exposure alters receptor-mediated signalling mechanisms, giving rise to a wide variety of effects [142]. The general disruption of cellular development caused by nicotine is also likely to contribute to altered synaptic function. Such synaptic hypoactivity has been demonstrated for catecholamines, both in the immediate postnatal period and into adolescence [93].

Prenatal nicotine also affects several neuromodulator systems, i.e. compounds that mainly act as modulators of synaptic transmission of other classical neurotransmitters. Such neuromodulators are e.g. substance P, which is well known to be involved in respiratory control, and galanin in central catecholaminergic pathways. Alterations in these systems may lead to a decreased respiratory response when subjected to stress situations [136,137]. These effects of nicotine may be of particular interest as deficiencies in neuromodulator systems, in contrast to similar deficits in classical neurotransmitter systems, may not be detected under basal conditions. However, it may be of vital importance when the organism is subjected to various types of stress and adequate responses are needed for survival.

Thus, the effects of nicotine on the developing nervous system causes disruption on multiple levels, ranging from cell damage and death to specific alterations of neural activity and disturbances in intercellular signalling mechanisms. All of these effects are likely contributing to the various disturbances seen after prenatal exposure to nicotine.

## 7. CONCLUSION

Several authors have previously pointed out that possible hazards of nicotine use during pregnancy must be placed in its context, i.e. that the alternative is smoking tobacco which is extremely harmful to both the woman and her child. In light of the limited amount of human data, comparison between the two has been difficult. However, in clinical praxis it has generally been viewed that while smokeless tobacco may not be less harmful than smoking, it is unlikely to be more hazardous. In this article, results from human and animal data are reviewed that indicate that for some parameters this may not be correct. Logically, nicotine replacement should be safer than smoking, but several animal studies indicate that the total dose of nicotine that the fetus is exposed to may be what really matters for brain development. Since conventional doses of NRT may be less effective in pregnancy, the higher nicotine doses needed may exceed the threshold for alterations in brain development and cause fetal harm. Similarly, longer acting nicotine replacement products leading to higher doses of total nicotine may thereby be more likely to contribute to these effects. Evidence from animal experiments indeed support this hypothesis, indicating postnatal behavioral and cognitive deficits in animals exposed in utero as well as molecular alterations affecting neurotransmitter function and neural development. These deficits last beyond the newborn period and at least into adolescence, thus representing a form of perinatal programming.

Knowledge about the possible adverse effects of prenatal nicotine exposure in humans is to date limited because use of smokeless tobacco is relatively uncommon in pregnant women, at least in the industrialized world. The few studies that exist on the subject are in most cases compromised by a lack of prospective recordings of the amount and timing of nicotine exposure during pregnancy. Also, the lack of objective measures of nicotine exposure (in e.g. urine or hair) decreases the validity of the studies. Furthermore, the numerous confounding environmental variables are problematic in interpreting results. Prospective studies of the effects of smokeless tobacco during pregnancy will provide new insight and increase our knowledge concerning the etiology of the well documented effects of smoking during pregnancy, as well as the existence of certain vulnerability periods. At present, the common conception that NRT is a better alternative than smoking during pregnancy may lead to an increased use of smokeless tobacco in both industrialized and developing countries. As recent findings indicate that functional nAChRs are present very early in neuronal development, and that activation at this stage leads to apoptosis and mitotic abnormalities, a total abstinence from all forms of nicotine should be advised to pregnant women for the entirety of gestation. In clinical praxis, this also highlights the importance of counseling and support in assisting pregnant women in altering their behavior.
